# Effectiveness of salivary stimulation using xylitol-malic acid tablets as coadjuvant treatment in patients with gastro-oesophageal reflux disease: early findings

**DOI:** 10.4317/medoral.23887

**Published:** 2020-10-09

**Authors:** Irene Sánchez-Blanco, Manuel Rodríguez-Téllez, José Ramón Corcuera-Flores, Carolina González-Blanco, Daniel Torres-Lagares, María Ángeles Serrera-Figallo, Guillermo Machuca-Portillo

**Affiliations:** 1 Associate Professor. Master Program of Special Care in Dentistry. University of Seville, Spain; 2 Associate Professor of Gastroenterology. Department of Internal Medicine. University of Seville, Spain; 3 Associate Professor. Master Program of Oral Surgery. University of Seville, Spain; 4 Full Professor. Department of Stomatology. University of Seville, Spain; 5 Associate Professor of Special Care in Dentistry. Department of Stomatology. University of Seville, Spain

## Abstract

**Background:**

Besides dental erosion syndrome, other oral syndromes could benefit from the stimulation of salivary secretion, in patients with gastro-oesophageal reflux disease (GORD). Our aims is evaluate the improvement of oral extra-oesophageal manifestations in patients with GORD using xylitol–malic acid tablets to stimulate salivary secretion.

**Material and Methods:**

The effectiveness of salivary stimulation using xylitol–malic acid tablets (as a supplement to omeprazole 40 mg/day) was assessed in a clinical trial (n = 14) lasting six months with patients with prior positive pH-metry, through GORD extra-oesophageal clinical signs, GerdQ and RDQ questionnaires, odontological variables, basal salivary secretion, stimulated salivary secretion, pH and buffer capacity, mucosal erythema index and dental wear. Statistics: chi-square (Haberman post-hoc), ANOVA, and Mann-Whitney U; variables between visits were evaluated with McNemar’s Student’s t and Wilcoxon tests; *p* < 0.05.

**Results:**

100% of patients not taking xylitol–malic acid presented xerostomia, but only 14.3% of patients taking xylitol–malic acid (*p* < 0.01) did. The mean saliva-buffer capacity at the last visit for patients not taking xylitol–malic acid was 2.14 ± 0.38, versus 2.71 ± 0.49 for patients taking xylitol–malic acid (*p* < 0.05). Retro-sternal burning (*p* < 0.05), heartburn (*p* < 0.05) and regurgitation (*p* < 0.05) were also reduced.

**Conclusions:**

Xylitol–malic acid tablets improve quality of life among patients with GORD, by reducing dry mouth, increasing saliva buffering and reducing heartburn, retro-sternal burning and regurgitation.

** Key words:**Tooth wear, erosion, gastroesophageal reflux, saliva.

## Introduction

Gastro-oesophageal reflux disease (GORD) is produced when reflux content from the stomach causes symptoms and/or complications (1). The reflux components that cause the most damage are acid and pepsin, although bile and pancreatic enzymes can also contribute to damage in some patients. GORD may or may not have typical symptoms, and it may manifest in patients with a normal oesophagus or with oesophagitis, but reflux episodes are abnormal in all cases (2).

Regarding the pathogenesis of GORD, many factors interact in different proportions, with the predominant factor varying by patient (e.g., defects in the anti-reflux barrier, defective oesophageal clearance, disorders of oesophageal mucosal resistance, increase in gastric acid secretion, delay in gastric emptying or circumstances in which intra-abdominal pressure increases) (1,3).

The typical reflux syndrome is characterized by the presence of heartburn and/or regurgitation. Heartburn is defined as a burning sensation in the retro-sternal area, while regurgitation is defined as perception in the mouth of or the hypopharynx of a flux from gastric content reflux. Apart from these 2 characteristic symptoms, patients may experience others, such as epigastric pain or difficulty falling asleep (1).

Lifestyle changes highly important from both the patient’s and the doctor’s perspective. Lifestyle changes can improve reflux symptoms in patients with slight to moderate GORD. Among these changes is diet control; avoiding food that can cause reflux is yet another therapeutic measure (4). Eating favours reflux episodes, eating slowly and going to bed at least 4 hours after meals appear to improve the clinical signs of GORD. Giving up smoking, losing weight, avoiding very tight clothing and raising one’s head during sleep are recommended (5,6). Lifestyle changes are often not enough; consequently, other therapeutic measures need to be resorted to (4).

A wide range of drugs are aimed at treating gastroesophageal reflux disease, such as alginates (sodium alginate), mucosal barrier protectors (sucralfate), prokinetic agents (metoclopramide, domperidone and cisapride), H2-histamine receptor antagonists (cimetidine, famotidine and ranitidine), proton-pump inhibitors (omeprazole, rabeprazole, pantoprazole, lansoprazole, esomeprazole, dexlansoprazole, and immediate-release omeprazole), TLESR reducers and transient lower oesophageal sphincter (LES) relaxation (baclofen) (7,8).

Laparoscopic Nissen fundoplication is the anti-reflux surgical treatment of choice (9). Surgery is indicated in case of medical therapy failure, an onset of complications derived from GORD such as peptic stenosis (10), intolerance to proton-pump inhibitors or patient preference and the desire for a permanent solution from patients who wish to relieve their symptoms without continuing pharmacological treatment for life (11).

Amongst the extra-oesophageal symptoms of GORD, those relating to the oral cavity take on an important dimension. Although researchers have mainly focussed on hard tissue lesions, some researchers have attempted to establish relationships with other oral manifestations and GORD (12). Amongst these oral manifestations are dental sensitivity, bitter taste (13), a burning sensation in the mouth and pharynx, mouth ulcers (3) and erythema on the palate and uvula (14,15). Few studies have been carried out on GORD’s role in tooth decay pathogenesis or periodontal disease, although Katunari (16) and Song *et al*. (17) detected the presence of pathological gingival changes in patients with GORD.

Other authors have focussed on the repercussions of salivary secretion on the oral state of patients with GORD because salivary secretion is a protective mechanism that helps to clear reflux acid from the oesophagus, thus reducing its harmful capacity on tissue (18). Several authors have studied the introduction of chewing gum into the normal treatment for GORD. Saliva production increases when the salivary gland is stimulated, which can compensate for low oral and oesophageal pH and prevent acid from reaching distal areas (19).

The aim of this study is to assess the improvement to oral extra-oesophageal manifestations in patients with GORD when using xylitol–malic acid Tablets as a coadjuvant for treatment along with omeprazole to stimulate salivary secretion.

Materials and Methods

This study was designed as an open, prospective, randomized pilot study to evaluate the effects of stimulating salivary secretion on oral manifestations in patients with GORD (confirmed using positive result pH-metric tests) (20). The inclusion criteria for the study were as follows: the patient must have at least 16 remaining teeth (including upper front teeth); have no other situations favouring reflux and dental erosions, specifically chronic alcoholism, anorexia or bulimia, or excessive consumption of acid foods; be between 18 and 70 years of age, and not be taking any anti-inflammatory or anticholinergic drugs. The presence or absence of dental erosions was not an exclusion factor.

Patients with GORD were randomly assigned using a random number assignation list (1:1) into two groups, to receive standard treatment of proton pump inhibitors (PPIs) or the experimental treatment of PPIs + saliva-stimulating Tablets. In all cases, double doses of omeprazole (40 mg) were administered once a day. This basic treatment was complemented by specific Tablets for stimulating salivary secretion, specifically of Xeros Dentaid® (Dentaid, Spain), indicated for treating dry mouth. Each patient received a box of individually packaged Xeros Dentaid® Tablets. They had to suck the Tablets until completely dissolved 3 times a day after main meals.

The Tablets were used as a coadjuvant treatment for GORD and its manifestations. The Tablet being placed in the patient’s mouth stimulates salivary secretion, swallowing and, with this, oesophageal clearance. Saliva may protect the oral cavity by diminishing the influx of gastric material, neutralizing acids and creating a protective barrier in the mouth. Xeros Dentaid® Tablets are part of a range of products indicated for treating dry mouth. The Tablets mainly comprise Xylitol (422.00 mg), an alcohol derived from xylose. Its composition includes a small proportion of malic acid (28.58 mg), whose function is to further stimulate saliva flow, and sodium fluoride (0.58 mg). Regular use of xylitol, a substance not fermenTable by oral bacteria, has been associated with decreased tooth decay, with lower counts of *S. mutans* and greater plaque pH (21).

The patients underwent regular check-ups to assess the progress of their oral parameters, with the final check-up being at six months. Initially, and for comparison between both study groups, the patients’ sex and age, medical history, service usage data and dental habits (time and reason for last visit to the dentist, frequency of visits to the dentist, frequency and type of brushing, frequency and type of interdental cleaning, use of mouthwash and use of prosthesis) were recorded.

Extra-oesophageal manifestations of GORD were recorded (chronic cough, laryngitis, pharyngitis, asthma, idiopathic pulmonary fibrosis, sinusitis, recurrent otitis media, chronic hoarseness and chronic bronchitis) at the study’s outset and after six months, as were other odontological variables (xerostomia, burning mouth, dysgeusia, basal and stimulated salivary secretion, saliva pH, buffer capacity (by adding HCl, with 1 = low, 2 = medium and 3 = high), O’Leary’s oral hygiene index, plaque index, calculus index, CAOD index, mucosal erythema index (with a score of 0 = normal, 1 = slight erythema, 2 = moderate erythema and 3 = severe erythema), ulcerations (0 = absent, 1 = ulcers less than 0.25 cm in diameter and 2 = ulcers are larger .25 cm in diameter) and dental wear (applying Smith and Knight’s dental wear index [TWI] (22), with a score of 0 to 4). Validated GerdQ questionnaires were also recorded (with each question scored on a scale of 0 to 3, with 0 = no episode in the last week, 1 = 1 episode in the last week, 2 = between 2 and 3 episodes in the last week and 3 = from 4 to 7 episodes in the last week), as was RDQ, scored on 2 dimensions: frequency (0 = never, 1 = less than once per week, 2 = once per week, 3 = between 2 and 3 times a week, 4 = from 4 to 6 times a week and 5 = daily episodes) and intensity (0 = no episodes, 1 = a very slight episode, 2 = a slight episode, 3 = a moderate episode, 4 = a moderate to severe episode and 5 = a severe episode).

This study was conducted in accordance with the ethical principles for medical research on humans, as defined in the Declaration of Helsinki, and following the standards of good clinical practice. Approval was obtained from the Ethical Committee of Virgen del Rocío and Virgen Macarena Hospital before commencing the study. Each patient was duly informed and required a signed informed consent form to take part in the study.

Statistical analysis was undertaken using the program SPSS 12.0. The Kolmogorov-Smirnov test was used to check for normality of the quantitative variables. Statistical differences between the two groups were compared with qualitative variables using the chi-square test and with the Haberman post-hoc test applied. For numerical variables, analysis of variance (ANOVA) was used when a normal distribution was present, and the Mann-Whitney U test was used with nonnormal distributions. To evaluate the progress of a variable over the different visits, the McNemar non-parametric test was used on dichotomous variables, Student’s t-tests were on numerical variables with normal distributions and the Wilcoxon test was used on numerical variables with nonnormal distributions and on ordinal qualitative variables. Values of *p* < 0.05 were deemed statistically significant.

## Results

Fourteen patients with GORD (Table 1) were studied: 10 women and 4 men. Of these patients, 9 were younger than 55 years of age (5 of these patients took Tablets), and 5 patients were over 55 years of age (2 took Tablets). In total, 7 (50%) took the Tablets and 7 (50%) did not.

Regarding the patients’ medical history (Table 2), 2 (28.6%) presented a history of heart failure, 2 (28.6%) presented a history of high blood pressure, 1 (14.3%) presented thyroid problems and 2 (28.6%) presented other pathologies. Four patients presented anxiety and/or depression, of whom 1 (7.1%) was not taking Tablets and 3 (21.4%) were taking them.

**Table 1:**
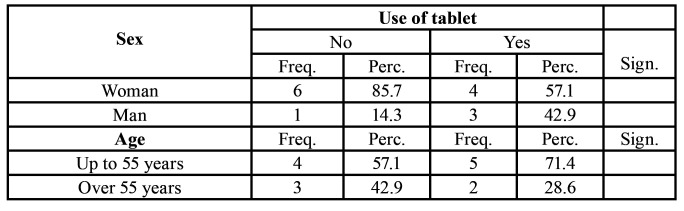
Initial data relating to age and sex in the sample studied.

**Table 2:**
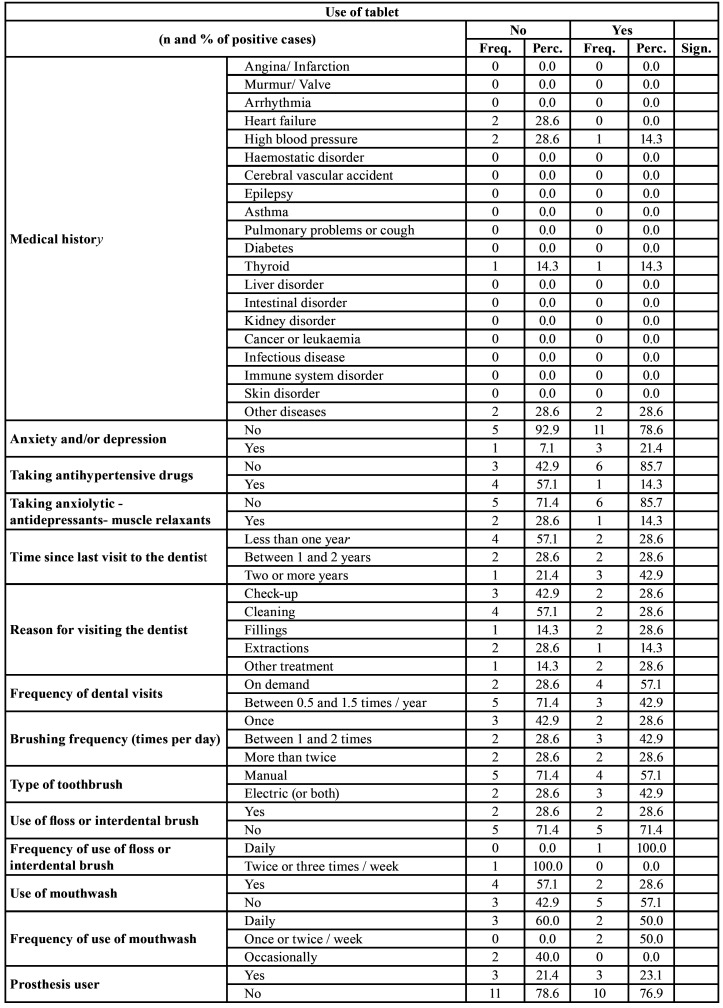
Medical history, oral habits and dental variables in the sample studied.

Regarding the time since their last visit to the dentist, among those who had last visited a dentist less than 1 year ago, 4 patients (57.1%) were not taking the Tablets, and 2 (28.6%) were. For those from whom it had been between and 1 and 2 years since the last visit, 2 (28.6% of patients) were not taking the Tablets, and 3 (42.9%) patients were. Regarding the reason for visiting the dentist, the most common one was for scaling and dental prophylaxis, with 4 (57.1%) of these patients not taking the Tablets and 2 (28.6%) who were.

The study groups were comparable, in terms of epidemiological and socioeconomic variables, since no significant differences were found.

Regarding the variables studied, the following data were found at the outset. With reference to extra-oesophageal manifestations (Table 3), chronic cough was presented by 1 (14.3%) patient who was not taking the Tablets and 4 (57.1%) patients who were (*p* < 0.05); with regard to chronic hoarseness, it was presented by 3 (42.9%) patients who were taking the Tablets (*p* < 0.05). The emergence of laryngitis, pharyngitis, asthma, pulmonary fibrosis, sinusitis or otitis media had no significant relationship with taking the xylitol–malic acid Tablets.

Regarding the dental history variables (Table 3), no significant differences were found between the patients taking xylitol–malic acid and those who were not taking it in terms of the variables identifying the presence of xerostomia, dysgeusia or burning mouth; those referring to pH, basal and stimulated saliva or saliva-buffering capacity; the indices for plaque, calculus, CAOD index or mucosal erythema index; number of ulcers; or tooth wear.

Regarding the GerdQ questionnaire, on average, the results for a burning sensation behind the sternum were 4.00 ± 0.00 for the group not taking the Tablets and 2.57 ± 1.27 for the patients who were taking the Tablets (*p* < 0.05). No significant differences were found between the study groups for any of the other aspects contemplated in this index (Table 3).

Regarding the RDQ (Frequency) index, significant differences (*p* < 0.01) between both groups were only found at the start of the study in relation to localized burning behind the sternum (6.00 ± 0.00 for patients not taking the Tablets versus 3.75 ± 2.07 for those who were), stomach acidity (6.00 ± 0.00 for patients not taking the Tablets versus 3.14 ± 2.27 for those who were) (*p* < 0.01) and gastric reflux (4.00 ± 2.24 for patients not taking the Tablets versus 1.43 ± 1.13 for patients who were) (*p* < 0.05) (Table 3).

In relation to the RDQ (Intensity) index, the 2 groups only had significant differences (*p* < 0.01) in connection with burning behind the sternum (5.29 ± 0.76 for patients not taking the Tablets versus 3.43 ± 2.07 for those who were), stomach acidity (5.29 ± 0.76 in patients not taking the Tablets versus 2.71 ± 1.89 for those who were taking xylitol–malic acid) (*p* < 0.01) and gastric reflux (3.29 ± 2.14 in patients not taking the Tablets versus 1.57 ± 1.51 in those who were taking xylitol–malic acid) (*p* < 0.05) (Table 3).

Between the first and last visit, after 6 months, both groups had similar initial values for their socioeconomic aspects and medical and odontological history. Significant differences were only found in some values for GerdQ and RDQ. Notwithstanding, this difference justified why the analysis of both groups regarding the studied values should not be carried against each the other; rather, we focused on the improvement in each group, taking as the first-visit values as the reference point.

No significant differences between the patients taking the xylitol–malic acid Tablets and the patients not taking them were established with respect to the majority of the studied variables, when the first-visit data were compared with the final-visit data (Table 4). No significant differences were found after 6 months in the extra-oesophageal or dental history manifestations, the CAOD index evaluation, the dental wear evaluation or the mucosal erythema index. Regarding saliva analysis, the group that did take the xylitol–malic acid Tablets had increased buffer capacity (up to 2.71 ± 0.49 from values starting at 2.17 ± 0.41), a statistically significant difference (*p* < 0.05), while the group that did not take the Tablets did not obtain any improvement in this parameter.

Regarding the GerdQ and RDQ questionnaires (Table 4), several symptoms showed improvements. The improvement to the burning sensation behind the sternum (GErdQ) was similar between the 2 groups, at the same percentage. At the end of the study, the group not taking the Tablets presented a mean of 50% of the first-visit value for this value, compared with 50.19% for the group taking the Tablets (*p* < 0.05 in both cases). Regarding the same sensation but measured with RDQ (Frequency) and RDQ (Intensity), the improvement in both groups was similar in both groups and statistically significant (*p* < 0.05). On the RDQ (Frequency) measurement, the mean value at week 3 was 42.83% of the initial one for the group not taking the Tablets, versus 40.05% for the other group. Similar improvement percentages were obtained on the RDQ (Intensity) for the same variable (*p* < 0.05).

The results for the stomach acidity variable in the RDQ (Frequency) and RDQ (Intensity) behaved similarly. Both study groups showed statistically significant improvements, with a similar intensity, when comparing data from the first and final visits (*p* < 0.05). The rest of the variables did not present any statistically significant differences between the first- and last-visit values (Table 4).

**Table 3:**
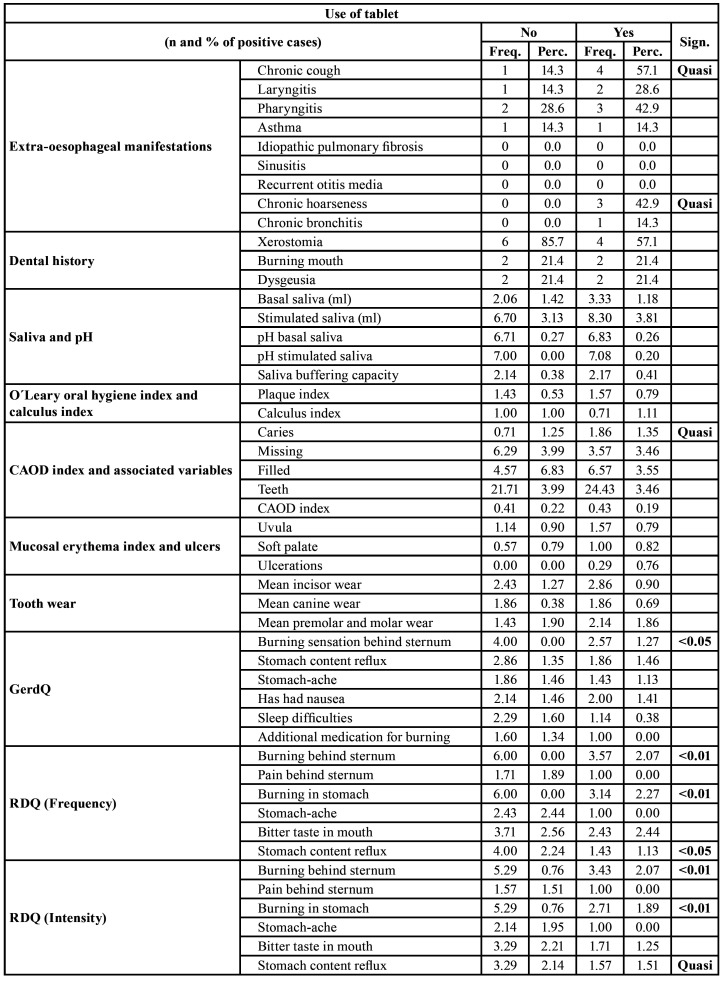
Extra-oesophageal manifestations.

**Table 4:**
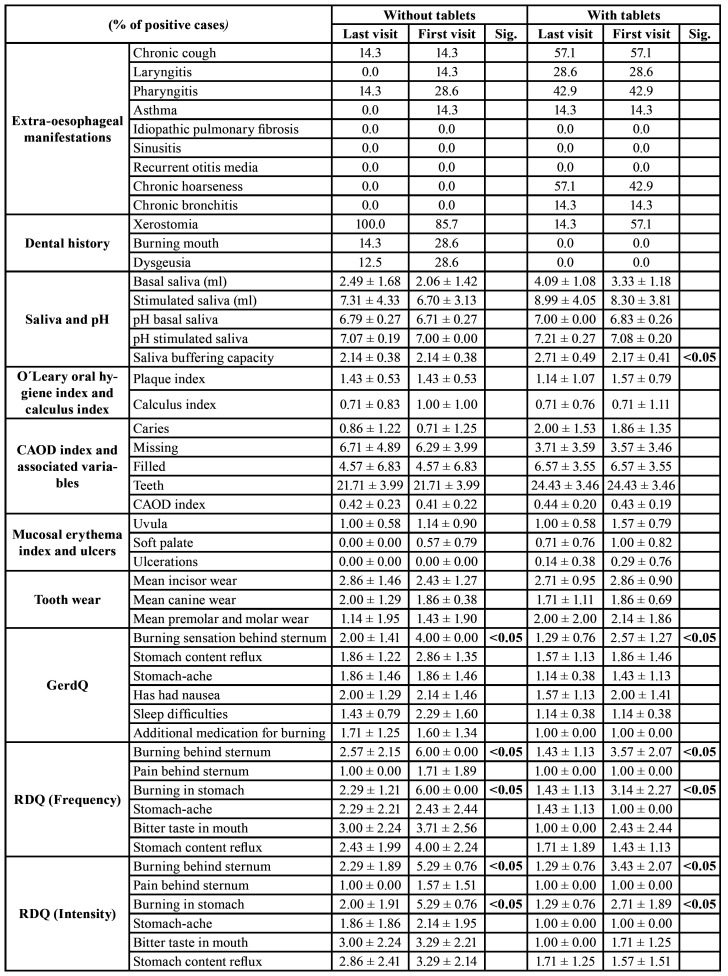
Comparison of variables between first and last visit in study groups.

## Discussion

The two groups had significant differences in terms of the xerostomia variable. Upon completing the study, all of the patients in the control group (100%) stated that they had suffered xerostomia, compared to only 2 (14.3%) in the group taking the Tablets. This finding implies that consumption of chewable Tablets increases salivary secretion.

Lapiedra *et al*. (23) reached the same conclusion in a study on 34 patients who were divided into 2 groups; 1 group was administered saliva-stimulating Tablets containing xylitol, and the other control group was provided sorbitol Tablets for 1 week. After resting for 1 week, the groups were inverted, with the study group receiving sorbitol Tablets and vice versa. In this study, xerostomia was measured using a subjective questionnaire, and both groups showed decreased xerostomia as compared with the start of the study, but only the study group taking xylitol Tablets had a significant decrease, as compared with the group taking sorbitol (23).

The difference between the work by Lapiedra *et al*. and the present study is that, in this study, the amount of saliva was collected and measured (both basal and stimulated) using paraffin pastilles, while in the study by Lapiedra *et al*., it was quantified using a questionnaire (23). Another difference between this study and the one carried out by Lapiedra *et al*. (23) was that in the latter, the authors administered 4 xylitol Tablets per day, compared to 3 in this study.

In another study by Peres *et al*. (24) undertaken on 27 patients, using chewable maltose Tablets resulted in increased compared with baseline measurements. Furthermore, 2 literature reviews carried out by Brosky *et al*. (25) and by Plemons (26) claim that chewable Tablets increase salivary secretion in patients with xerostomia, thus improving their quality of life.

The present study also observed that the saliva-buffering capacity increased significantly on the final visit among patients treated with Tablets, compared to those who were not treated with them. One can deduce that the Tablets not only increased the amount of saliva but also improved its chemical properties and acid-neutralizing buffer capacity.

This finding corroborates the conclusions of Kharevich *et al*. (27), who, in a pilot study on 10 patients, also observed increased saliva-buffering capacity, which reduced its acidity and minimised xerostomia, as in this study with xylitol–malic acid Tablets.

The present study also found some improvement among the patients treated with Tablets with respect to bitter taste in the mouth because those who did not take the xylitol–malic acid Tablets experienced bitter taste more intensely and more frequently. Note that bitter taste in the mouth is one of the most characteristic symptoms of GORD, (28,29) so much so that although certain drugs objectively improve the disease’s symptoms (such as proton pump inhibitors), they do not prevent patients from experiencing a bitter taste in their mouth, with this symptom persisting despite the treatment (30). For this reason, it is very important that the xylitol–malic acid Tablets used in this study relieved this unpleasant extra-oesophageal symptom of GORD.

Summarising the results provided by this study, xylitol–malic acid Tablets (Xeros®) demonstrated the capacity to improve quality of life in patients with GORD by reducing xerostomia, improving saliva-buffering capacity and relieving regurgitation and the sensation of gastric and retrosternal burning.
